# Letter to Editor on: "Ginger (*Zingiber officinale* roscoe) extract could upregulate the renal expression of NRF2 and TNFα and prevents ethanol-induced toxicity in rat kidney" by Akbari et al.

**DOI:** 10.22038/AJP.2022.21187

**Published:** 2023

**Authors:** Perumal Elumalai, Devaraj Ezhilarasan, Subramanian Raghunandhakumar

**Affiliations:** *Department of Pharmacology, The Gold lab, Molecular Medicine and Toxicology Division, Saveetha Dental College, Saveetha Institute of Medical and Technical Sciences (SIMATS), Chennai, Tamil Nadu, India*

**Keywords:** Zingiber officinale, Ginger, Ethanol, Nuclear factor E2-related factor

**Dear editor**

A recent study entitled “Ginger (*Zingiber officinale* roscoe) extract could upregulate the renal expression of Nrf2 and TNFα and prevents ethanol-induced toxicity in rat kidney” that was published in this journal by Fathi et al., 2021) states that ethanol can activate Nrf2 expression. Nuclear factor E2-related factor (Nrf2) regulates the expression of a variety of genes responsible for intracellular detoxification, and antioxidant, anti-inflammation, anti-stress activities, etc (Ezhilarasan et al., 2016 ▶). Nrf2 is crucial for the induction of endogenous antioxidant enzymes such as heme oxygenase 1 and reduced glutathione via activation of intracellular antioxidant responsive element. Therefore, activation of Nrf2 pathway by phytochemicals has been demonstrated to provide protection against alcohol-related abnormalities suggesting the beneficial role of Nrf2 activation (Zhao et al., 2018 ▶; Ezhilarasan et al., 2016 ▶). Besides, a growing body of evidence reports the protective role of Nrf2 against a number of kidney diseases (Hejazian et al., 2021 ▶). Nrf2 activates several intracellular antioxidant enzymes during the onset of intracellular oxidative stress (Ezhilarasan, 2018 ▶). Therefore, Nrf-2 activation is usually considered to have a beneficial effect. Previous studies also showed that ethanol administrations can downregulate or suppress Nrf-2 expression (Sueblinvong et al., 2014 ▶; Shanmugam et al., 2019 ▶). In view of the above scenario, as a powerful oxidative agent, how ethanol could activate Nrf2 expression? The authors have showed that ginger, ethanol and ethanol+ginger treatments can activate Nrf2 expression. The manuscript was also failed to discuss about the reason behind Nrf2 upregulation after ethanol administration. These discrepancies must be addressed in the discussion part.

The objectives of the study were interesting, however, there were some discrepancies in the title and their results furnished. Therefore, we wish to seek your attention towards this publication. The main concern of this publication is the title. The title says “Ginger (*Zingiber officinale* roscoe) extract could upregulate the renal expression of Nrf-2 and tumor necrosis factor alpha (TNF-α) and prevents ethanol-induced toxicity in rat kidney”. However, in results the authors were reported that Ginger can downregulate ethanol-induced TNF-α. Moreover, as reported in the title Ginger extract did not ameliorate ethanol-induced Nrf-2 downregulation. 

## Conflict of interest

The author declares that he has no conflicts of interest to disclose. 
